# Anti-hyperalgesic effects of photobiomodulation therapy (904 nm) on streptozotocin-induced diabetic neuropathy imply MAPK pathway and calcium dynamics modulation

**DOI:** 10.1038/s41598-022-19947-2

**Published:** 2022-10-06

**Authors:** Willians Fernando Vieira, Kauê Franco Malange, Silviane Fernandes de Magalhães, Júlia Borges Paes Lemes, Gilson Gonçalves dos Santos, Catarine Massucato Nishijima, Alexandre Leite Rodrigues de Oliveira, Maria Alice da Cruz-Höfling, Cláudia Herrera Tambeli, Carlos Amilcar Parada

**Affiliations:** Department of Structural and Functional Biology, Institute of Biology, University of Campinas (UNICAMP), Carl von Linnaeus n/n, Cidade Universitária Zeferino Vaz, Campinas, SP 13083-864 Brazil

**Keywords:** Ion channels in the nervous system, Molecular neuroscience, Neuronal physiology, Peripheral nervous system, Neurology

## Abstract

Several recent studies have established the efficacy of photobiomodulation therapy (PBMT) in painful clinical conditions. Diabetic neuropathy (DN) can be related to activating mitogen-activated protein kinases (MAPK), such as p38, in the peripheral nerve. MAPK pathway is activated in response to extracellular stimuli, including interleukins TNF-α and IL-1β. We verified the pain relief potential of PBMT in streptozotocin (STZ)-induced diabetic neuropathic rats and its influence on the MAPK pathway regulation and calcium (Ca^2+^) dynamics. We then observed that PBMT applied to the L4-L5 dorsal root ganglion (DRG) region reduced the intensity of hyperalgesia, decreased TNF-α and IL-1β levels, and p38-MAPK mRNA expression in DRG of diabetic neuropathic rats. DN induced the activation of phosphorylated p38 (p-38) MAPK co-localized with TRPV1^+^ neurons; PBMT partially prevented p-38 activation. DN was related to an increase of p38-MAPK expression due to proinflammatory interleukins, and the PBMT (904 nm) treatment counteracted this condition. Also, the sensitization of DRG neurons by the hyperglycemic condition demonstrated during the Ca^2+^ dynamics was reduced by PBMT, contributing to its anti-hyperalgesic effects.

## Introduction

Diabetes can damage the peripheral nervous system (PNS) in various ways, and diabetic neuropathy (DN) is one of the most common complications of untreated diabetes^1^. DN is a chronic complex disorder that affects the peripheral nerves, causing a painful condition involving superior and inferior limbs^1–4^ with an incidence rate of about 70% of diabetic patients^5,6^. The mechanism by which hyperglycemia leads to peripheral nerve injury is not very clear, but it is known that several metabolic pathways are affected^7^. The main events involve the polyol pathway, through the aldose reductase (AR) activation^8–11^, the protein glycosylation, and the advanced glycation end-products (AGEs) production^10,12,13^. In addition, the formation of free radicals linked to oxidative stress^14,15^, the reduced neurotrophic support^16,17^, and the increased protein kinase C activation (PKC)^9,18^ contribute to the peripheral damage. As a result of the metabolic imbalance, mitochondrial failure^10,19,20^ and inflammatory processes are also frequent and related to the phosphorylation of mitogen-activated protein kinases (MAPKs)^21–25^.

MAPK is a family of serine/threonine protein kinases responsible for transducing extracellular stimuli into intracellular posttranslational and transcriptional responses^26–28^. It comprises p38-MAPK, extracellular signal-regulated protein kinase (ERK1/2), and c-Jun N-terminal kinase/stress-activated protein kinase (SAPK/JNK)^29^. The three main subfamilies of MAPK (p38, ERK1/2, and JNK) coordinate several functions: gene transcription, protein synthesis, cell cycle, proliferation, differentiation, and apoptosis^30–33^. Extracellular stimuli, such as proinflammatory cytokines^34–36^ and oxidative stress^30,37^ can activate the MAPK pathway, also under influence of Ca^2+^ dynamics^38,39^. Membrane depolarization can promote calcium influx through L-type calcium channels and activates MEK1, which phosphorylates MAPK^39^. Hyperglycemia seems to be one of the factors which could stimulate the MAPKs phosphorylation^40,41^, once the activation of p38 has been seen in peripheral sensory neurons of diabetic rats^42^, specifically in the dorsal root ganglia (DRG)^43–46^. Similarly, the phosphorylation of JNK leads to apoptosis of hyperglycemia-stressed neurons via activation of caspase-3^22,47–49^. Furthermore, MAPK signaling stimulates the transient receptor potential vanilloid subtype 1 (TRPV1) expression in chronic pain, a highly Ca^2+^-permeable channel^50^. This implicates several diabetes complications, including thermal hyperalgesia^51^.

Owing to the complexity of the metabolic alterations observed in DN, there are several pharmacological targets for treating the painful condition, but with low efficacy. Most patients refer to some relief of the symptoms, but it regresses over time, even before treatment ends^52^. Characterized as a nonthermal process, photobiomodulation therapy (PBMT) involves the activation of specific cellular chromophores, such as cytochrome *c* oxidase (CCO; mitochondrial complex IV) with red and infrared irradiation^53^. This process is triggered by photophysical and photochemical reactions inside the cells when the light crosses the cell membrane^54–56^, causing the modulation of specific pathways related to cellular survival, such as the increase in adenosine triphosphate (ATP), oxygen production, and nitric oxide (NO) release^57^. As a result, photobiomodulation can be used as a therapy to treat some painful conditions^58–65^ including DN, at least complementary, as observed in a previous study conducted by our group^66^. Based on that, this study aimed to analyze the anti-hyperalgesic effects of PBMT (904 nm) on streptozotocin (STZ)-induced diabetic neuropathy, considering the role of the MAPK pathway in the course of the disease and as a target for the PBMT mechanism.

## Results and discussion

### PBMT did not alter the metabolic parameters linked to STZ-induced type-1 diabetes (T1D) since its clinical signals (hyperglycemia, weight loss, polyuria, polydipsia, and polyphagia) in the STZ-PBMT group remain unaltered

**Figure 1:**
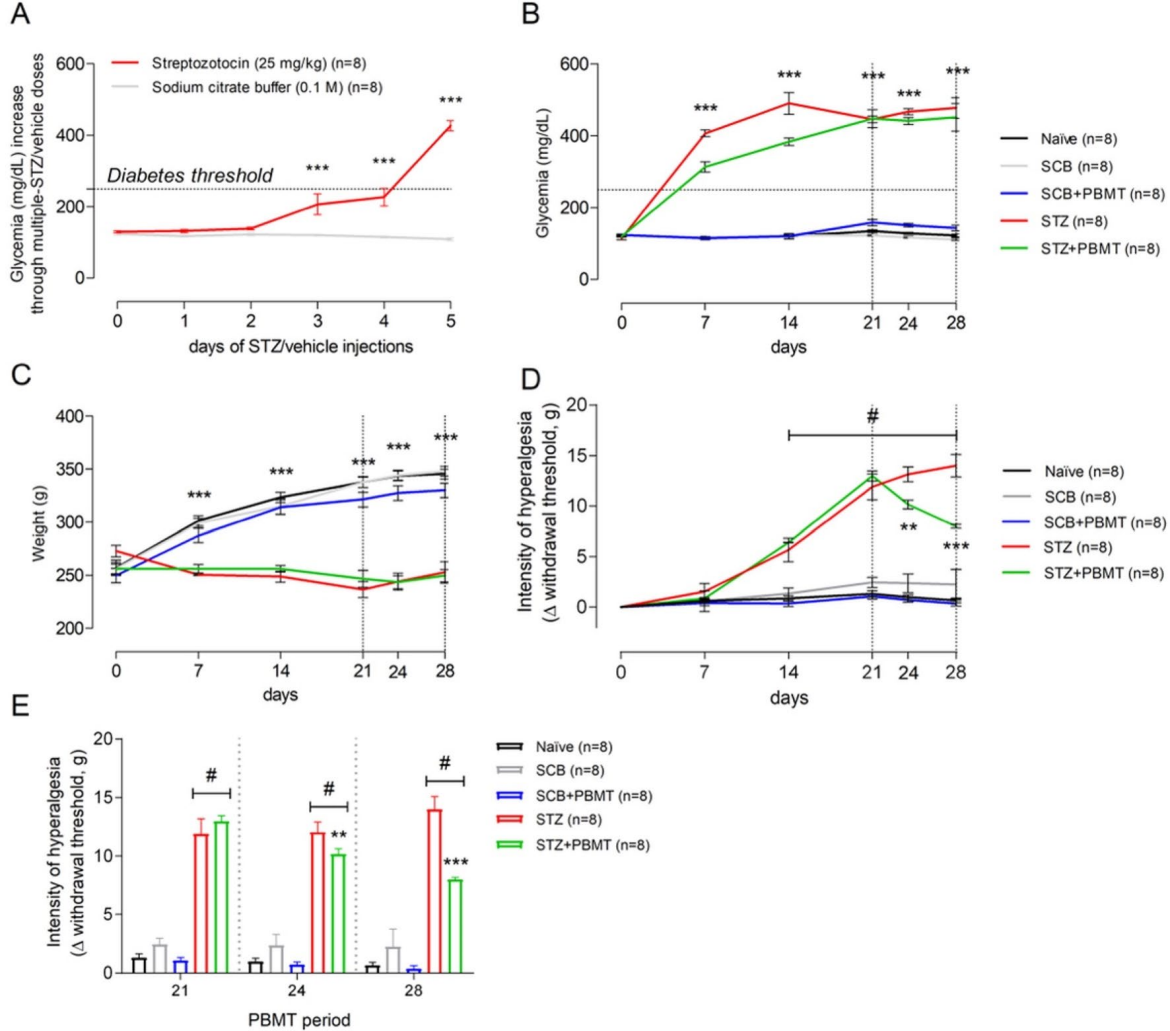
PBMT decreases the hyperalgesia of T1D rats without changing their glycemia or weight. (**A**) Average morning glycemia (red line) from rats during the protocol of T1D induction by multiple STZ low-doses; dotted horizontal black line: established diabetes threshold (glucose ≥ 250 mg/dL). (**B**) Rats from STZ (red line) and STZ + PBMT (green line) groups showed high levels of hyperglycemia at 7, 14, 21, 24, and 28 days. (**C**) Rats from STZ (red line) and STZ + PBMT (green line) groups stopped gaining weight after the installation of diabetes. (**D**) Data from mechanical withdrawal thresholds (Δ; g; the intensity of hyperalgesia) show that PBMT reduced significantly the intensity of hyperalgesia of the STZ + PBMT group (green line) in comparison with the STZ group (red line), at the 24th and the 28th days. (**E**) Bar graph emphasizing the PBMT anti-hyperalgesic effect during the period comprised between the 21st and the 28th days. In (**A**–**C**), symbol (***) means a significant difference (p < 0.001) between diabetic groups and controls (Two-way ANOVA followed by Bonferroni posthoc test). In (**D**) and (**E**), symbols (**) and (***) mean significant difference (p < 0.01 and p < 0.001, respectively) between STZ and STZ + PBMT groups (Two-way ANOVA followed by Bonferroni posthoc test); symbol (#) means significant difference (p < 0.001) between STZ-induced groups in comparison to control groups (Two-way ANOVA followed by Bonferroni posthoc test [**D**]; One-way ANOVA followed by Bonferroni posthoc test [**E**]). Data are expressed as mean ± S.E.M.; vertical dotted black lines indicate the PBMT period.

T1D induction protocol through low doses of STZ (five low doses, a single dose of 25 mg/kg per day) was suitable for the installation of irreversible hyperglycemia. All rats submitted to STZ injections (STZ and STZ + PBMT groups) became hyperglycemic (≥ 250 mg/dL of blood glucose concentration; 349.07 ± 48.23 mg/dL) after five STZ-low doses, reaching the threshold for hyperglycemia between the fourth and fifth days (Fig. 1A,B). Hyperglycemic rats also presented polyuria, polyphagia, and polydipsia (data not shown). They stopped gaining weight, with a slight weight loss (in grams) throughout the experimental period (Fig. 1C). The systemic administration of sodium citrate buffer (SCB), the STZ vehicle, did not alter the glycemia or the weight gain compared with control naïve rats.

STZ is a diabetogenic antibiotic known for its selective capacity to kill the pancreatic beta cells (β-cells), commonly used for the T1D model in rats^67–70^. STZ is taken up by the β-cells glucose transporter GLUT2 and triggers immune mechanisms^68,69^. According to Wang and Gleichmann^68,69^, STZ restricts GLUT2 expression in vivo and in vitro when administered through multiple low-doses protocols, which is a method that aims to produce fewer STZ side effects, such as neurotoxicity. In general, rats submitted to STZ injections show deficits in insulin production, leading to hyperglycemia and, consequently, polydipsia and polyuria^70^. All those diabetes signals were observed in rats submitted to the low-doses STZ protocol (STZ and STZ + PBMT groups), thus characterizing a reproducible model of diabetes induction, as shown in previous studies from our group^66,71–74^.

Diabetic rats, submitted or not to PBMT (STZ + PBMT and STZ groups, respectively), showed similar levels of hyperglycemia on days 21, 24, and 28 of the experimental protocol (454.94 ± 37.10 mg/dL). The same was observed regarding the rats' weight, once this parameter was only dependent on the diabetic condition and not on the PBMT. In like manner, PBMT did not cause any influence over healthy rats (control, non-diabetic and non-hyperalgesic; SCB + PBMT group), as shown in Fig. 1B,C. Our results corroborate with the study performed by Peplow and colleagues^75^, in which PBMT (660 nm; 100 mW; 4.7–6.3 J/cm^2^; 20 s) applied for wound healing in diabetic patients does not change the hyperglycemia or the patients' metabolic status.

### PBMT reduced STZ-induced diabetic hyperalgesia

The data reproduced previous results obtained from our research group^66^. On the 21st day (after the first PBMT session), there was no reduction in the mechanical hyperalgesia intensity (Δ withdrawal threshold, g). However, on the 24th and 28th days, a time-significant reduction (p < 0.01 and p < 0.001, respectively) was observed in the intensity of hyperalgesia in the STZ + PBMT group compared to the STZ group. Nonetheless, when PBMT was applied to control rats (SCB + PBMT group), there was no change in the mechanical withdrawal thresholds in a similar condition observed in SCB (vehicle) and Naïve groups, as shown in Fig. 1D,E (the latest in detail for the PBMT period).

PBMT has long been used for the clinical treatment of neuropathic pain with satisfactory results^76,77^, but its analgesic mechanisms are not entirely understood. The inhibition of the neuronal hyperactivity by the infrared light seems to be one of the ways that PBMT acts directly over the neurons^78,79^, and consequently over the pain outcomes. It has been suggested a neuromodulation effect because patients with back pain submitted to PBMT (808 nm; 100 mW; 8.4 J; 84 s; a single session) on L4-L5 levels present a significant pain relief^52,80^. This modulatory effect could be the key to regulating the neuronal activity affected by hyperglycemia avoiding painful sensibility.

### Motor function dysfunction related to DN and detected by the CatWalk system was amended by PBMT, likely due to PBMT's anti-hyperalgesic property

**Figure 2:**
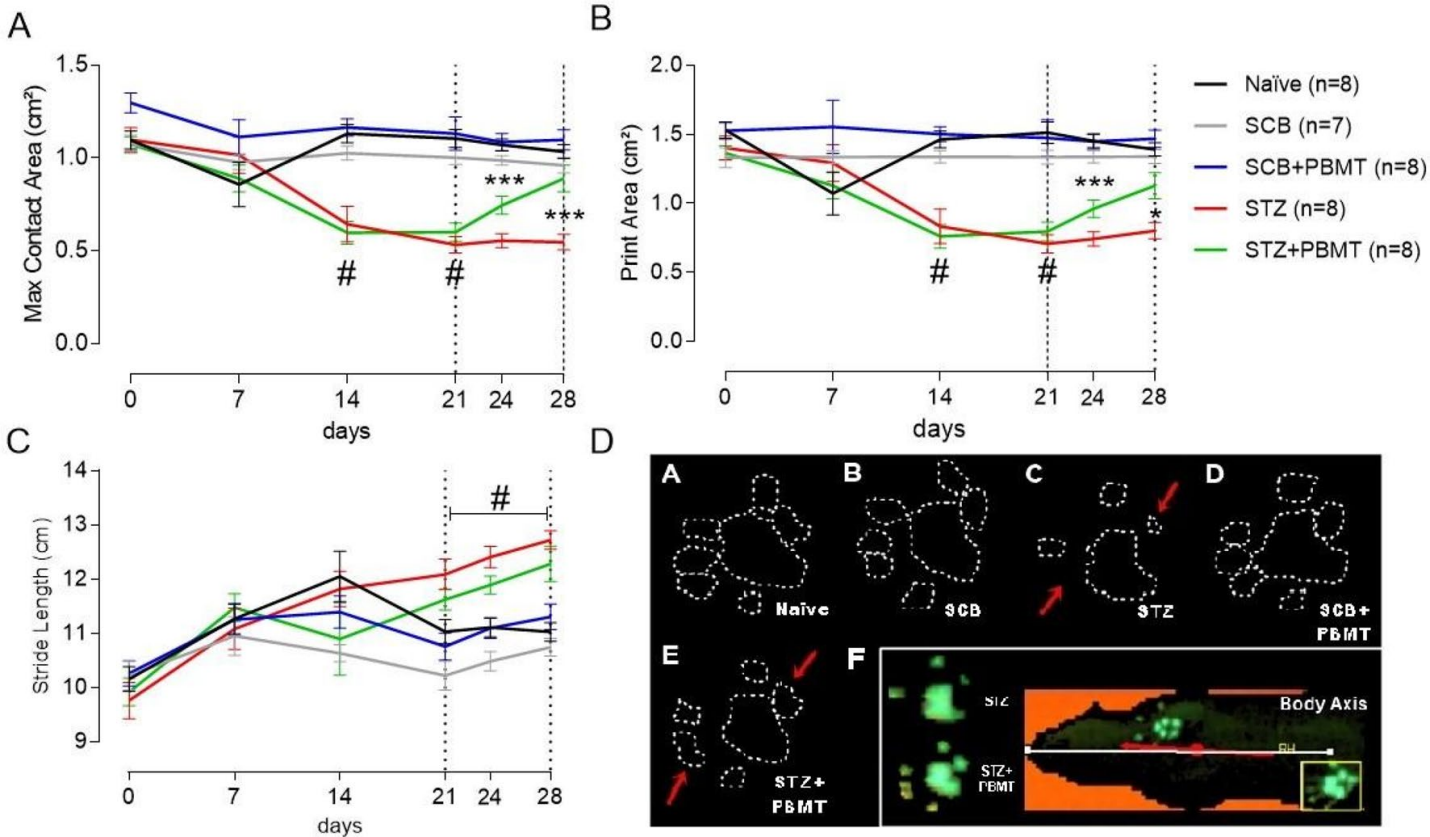
Gait spatial parameters were altered in DN and thwarted by PBMT. (**A**) Maximum Contact Area (cm^2^). (**B**) Print Area (cm^2^). (**C**) Stride Length (cm). Data are expressed as mean ± S.E.M.; symbols (*) and (***) mean significant difference (p < 0.05 and p < 0.001, respectively) between STZ and STZ + PBMT groups; symbol (#) means significant difference (p < 0.001) between STZ-induced groups (STZ and STZ + PBMT) in comparison to control groups (Naïve; SCB; SCB + PBMT); Two-way ANOVA followed by Bonferroni posthoc test. (**D**) The general pattern of the hind paw 2D footprints; rats from STZ group (c) showed a reduction in the contact area, delimited by the white dotted line and indicated by the red arrows; rats from STZ + PBMT group (e) presented a footprint area closest to the control groups (red arrows). In the lower right frame (f), *Body Axis* serves as a reference to observe the positioning of the paw during the analysis.

Our analysis was based on a previous study identifying dynamic motor function alterations related to STZ-induced DN^74^. As shown in Fig. 2A,B, PBMT was able to improve the Maximum Contact Area (cm^2^) and the Print Area (cm^2^) of the rats' hind paws after 4 (24th day) and 8 (28th day) PBMT sessions. Likely, these data suggest an analgesia amelioration ought to improve nerve conduction. Statistical differences were observed between STZ + PBMT and STZ groups in such periods. No difference, however, was observed for the Stride Length (cm) parameter (Fig. 2C), except between both neuropathic groups (STZ and STZ + PBMT) vs. control groups (Naïve, SCB, and SCB + PBMT). Areas of the digits and plantar pad (glabrous) appeared well delimited in the footprints of the right hind paw (RH) from rats of the STZ + PBMT group on days 24th and 28th (Fig. 2D: e), which were similar to the observed in Naïve and SCB groups (Fig. 2D: a, b, d), thus differing from the STZ group (Fig. 2D: c). The latest showed a reduction in the contact area with a low resolution of the footprints on the 28th day.

According to Zochodne et al.^81^, non-controlled diabetes leads to damage of sensory neurons before the involvement of the motor ones. DN is associated with postural changes in this process, involving the alteration of foot positioning during gait^82^ and alterations in pressure applied throughout the stand phase^83,84^. Our group demonstrated that Maximum Intensity (a. u.) was the main altered parameter on the 14th day after starting STZ injections^74^, which agrees with previous results by other groups^84^. Diabetic animals, which presented a decrease in the mechanical threshold on the 14th day, applied less pressure during the paws' contact with the glass floor of the CatWalk XT system. These findings follow the pattern of sensory changes in DN, also known as "stocking-and-glove," which occurs as a sensory disorder of the extremities of limbs, compromising functionality^2,85^.

Few studies evaluated the influence of PBMT on motor parameters in animal models, primarily when the CatWalk XT system was used. Snake venom-induced local myonecrosis provokes a semi-inflected posture of the affected hind limb 3 h after the venom injection. However, PBMT applied at the same period using the GaAs laser (904 nm; 4 J/cm^2^) returns the Maximum Intensity (u. a.), Stand (s), and Balance (s) to values similar to control^60^. Although hyperglycemic rats did not present a hind limb semi-flexion, they applied less pressure (represented by less intensity and smaller paw contact area) during gait. Therefore, PBMT could normalize, at least in part, the altered gait of hyperglycemic rats as a consequence of an anti-hyperalgesic effect promoted by this therapy. Altogether, it is plausible to suggest that diabetes-derived neuropathic pain likely results from impairment in sensory and motor neurons.

### PBMT reduced the levels of cytokines in DRG

**Figure 3:**
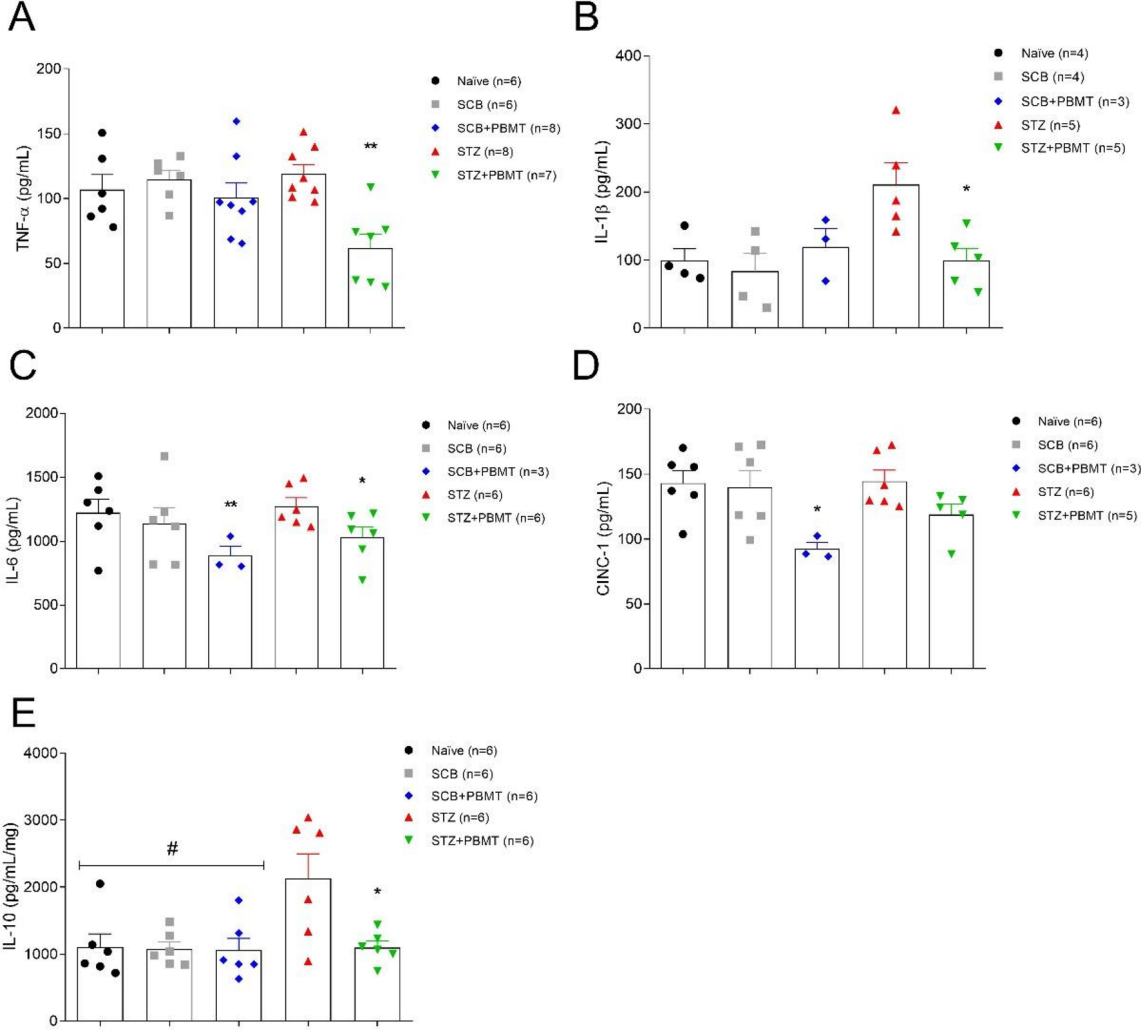
Effects of PBMT over the DRG levels of TNF-α, IL-1β, IL-6, CINC-1, and IL-10. (**A**) There was no increase in the levels of TNF-α (pg/mL) in the STZ group (red symbols); the STZ + PBMT group (green symbols) showed a significant reduction in the levels of TNF-α (pg/mL) in comparison to all the other groups. (**B**) For the levels of IL-1β (pg/mL), there was a significant increase in the STZ group (red symbols) in comparison with all the other groups. (**C**) Levels of IL-6 (pg/mL) were reduced significantly in SCB + PBMT (blue symbols) and STZ + PBMT (green symbols) groups, in comparison to all the other groups. (**D**) CINC-1 concentrations (pg/mL) showed a significant reduction in the SCB + PBMT group (blue symbols) only. (**E**) Levels of IL-10 (pg/mL/mg) showed a significant increase in the STZ group (red symbols) in comparison to all the other groups. Data are expressed as mean ± S.E.M.; symbols (*) and (**) mean p < 0.05 and p < 0.01, respectively; symbol (#) means that all control groups are significantly different from the STZ group (p < 0.05); One-way ANOVA followed by Bonferroni posthoc test.

To further investigate whether the anti-hyperalgesic effect of PBMT could be associated with reducing of cytokines in DRG, we analyzed the concentrations of TNF-α, IL-1β, IL-6, CINC-1, and IL-10 by ELISA immunoassay. As shown in Fig. 3B,E, the hyperglycemia increased the level of IL-1β and IL-10, respectively, while did not affect TNF-α, IL-6, and CINC-1 (panels A, C, and D, respectively). However, the PBMT decreased all analyzed cytokines independently of the hyperglycemia (Fig. 3). Agreeing with our findings, hyperglycemia especially increases the level of the pro-inflammatory cytokine IL-1 β and the anti-inflammatory cytokine IL-10 in DRG^10^. Also, the levels of TNF-α, IL-6, and CINC-1 were not affected by the hyperglycemia, suggesting any inflammatory background pure in DN. Nevertheless, this study did not analyze other inflammatory parameters, such as polymorphonuclear cell migration. However, data from this study demonstrated that PBMT decreased the level of cytokines in DRG, including the ones increased by hyperglycemia.

Although the pathophysiology of neuropathic pain depends on each type of neuropathy, the use of PBMT (660 nm; 30 mW; 9 J/cm^2^; 60 s; 7 consecutive days; 63 J/cm^2^ cumulative energy density) in hyperalgesia induced by sciatic nerve constriction model causes a reduction in the proinflammatory cytokines, such as TNF-α, IL-1β, and HIF-1α (hypoxia-inducible factor-1α)^86^. Similarly, it was described a reduction in TNF-α levels by PBMT (950 nm; 2.5 J/cm^2^; 32 s; 15 consecutive days; 37.5 J/cm^2^ cumulative energy density) in mice model of sciatic nerve crush injury^87^. These shreds of evidence suggest the anti-inflammatory capacity of PBMT. In this regard, the anti-inflammatory effects promoted by PBMT were previously observed in a carrageenan-induced inflammation model, in which both 660 nm and 684 nm low-level lasers (30 mW; 7.5 J/cm^2^; 196 s; a single point) reduced the edema formation and inflammatory cell migration at 4 h after carrageenan injections^88^.

Inflammatory cytokines can activate diverse cell membrane receptors, thus transmitting environmental signals, and in several cell lines, activation of G-protein-coupled-receptors (GPCRs) and receptor tyrosine kinases (RTKs) activate MAPKs^89^. MAPK cascades signaling are evolutionarily conserved pathways related to intracellular signal transduction in response to various extracellular stimuli^90^. MAPK controls many cellular processes, such as growth, proliferation, differentiation, motility, stress response, survival, and apoptosis^91^. All three groups of MAPK (p38; ERK1/2; JNK) can be activated by osmotic perturbations derived from glucose, polyol pathway, oxidative stress, and advanced glycation end products (AGE)^92^. These events are highly involved in the etiology of DN^1^.

### PBMT reverses the STZ-increased p38 MAPK gene- and protein expression

To examine a possible interaction between the inflammation and MAPK pathway, we further assessed MAPKs gene- and protein expression in L4-L5 DRG, once these molecular targets are supposed to be altered in DN, due to a condition imposed by the uncontrolled diabetes^92^. Before the real-time quantitative PCR experiments, primers for target- (p38, ERK1/2, and JNK) and housekeeping genes (endogenous controls: Arfgef1 and Serpinb6) were tested for their efficacy by the standard curve construction. It was done through serial dilutions of naïve cDNA (1:4; 1:8; 1:16; 1:32; 1:64) and a fixed primer concentration (100 nM). High efficiency was observed for all tested primers, including the endogenous controls (99.9; r^2^ 0.98) (data not shown). Then, the expression of target genes was normalized by the mean values of Ct (cycle threshold) from a pool of Arfgef1 and Serpinb6 gene expression.

**Figure 4:**
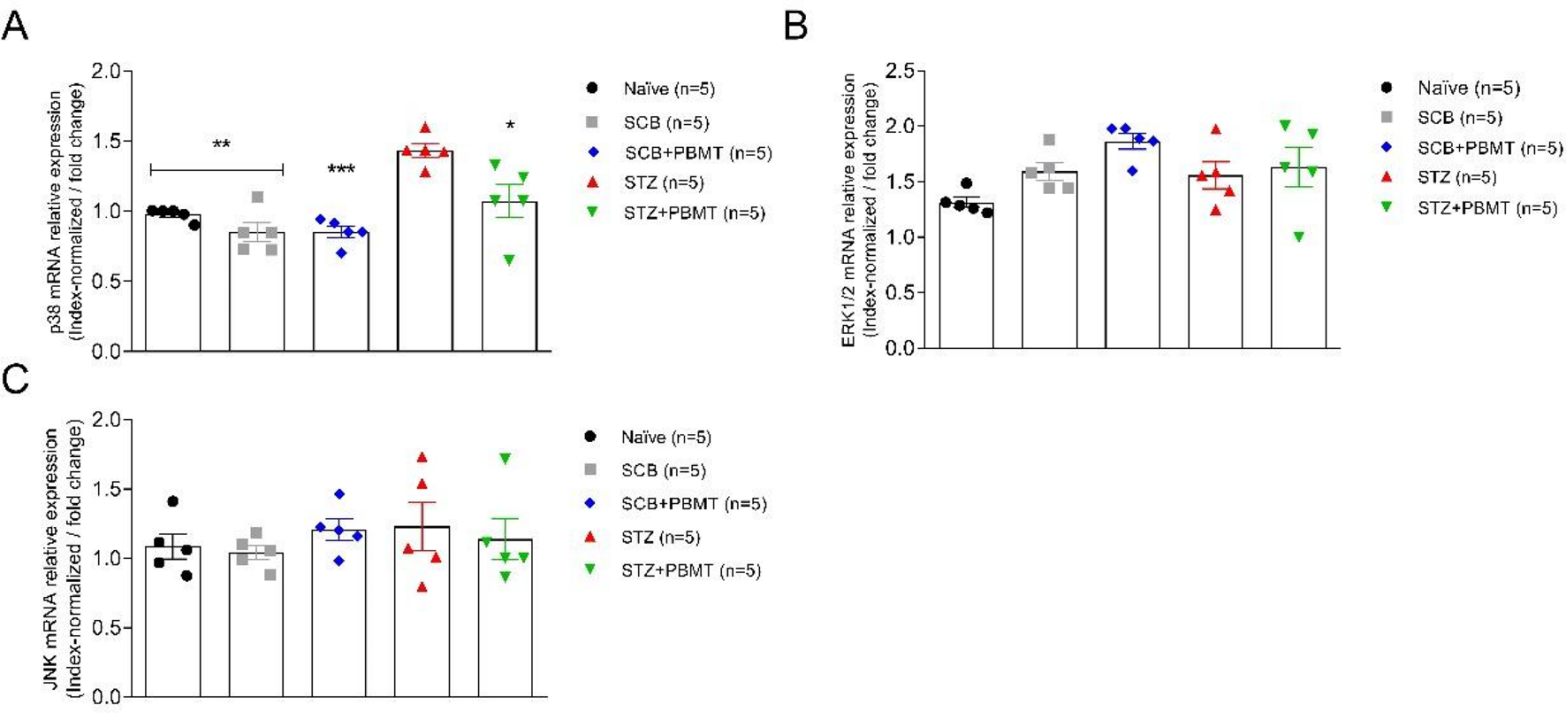
Quantitative expression of MAPK mRNA. Gene expression values of p38 (**A**), ERK1/2 (**B**), and JNK (**C**) were index-normalized by a pool of endogenous controls (Arfgef1 and Serpinb6) expression. PBMT decreased the p38 mRNA expression in hyperalgesic rats (STZ + PBMT group). Hyperglycemia increased p38 mRNA expression, but not ERK1/2 and JNK in DRG associated with hyperalgesia. Data are expressed as mean ± S.E.M.; symbols (*), (**), and (***) mean p < 0.05, p < 0.01, and p < 0.001 (respectively) in the comparisons between STZ (red symbols) and the other groups (Naïve, black symbols; SCB, gray symbols; SCB + PBMT, blue symbols; STZ + PBMT, green symbols); unpaired Student *t-*test.

In this study, we observed a significant increase in the expression of p38 mRNA in the STZ group compared to Naïve, SCB (vehicle) (p < 0.01), and SCB + PBMT groups (p < 0.001; unpaired Student *t-test*). In addition, p38 mRNA expression in the STZ group was significantly higher in comparison to the STZ + PBMT group (p < 0.05; unpaired Student *t-test*), as shown in Fig. 4A. Once PBMT does not modify glycemia, we conclude that this effect is associated with hyperalgesia, evident only in the STZ group. No differences in p38 mRNA were observed among the groups STZ + PBMT, SCB, and Naïve. Slight differences between the groups regarding ERK1/2 and JNK mRNA expression were not significant (Fig. 4B,C).

**Figure 5:**
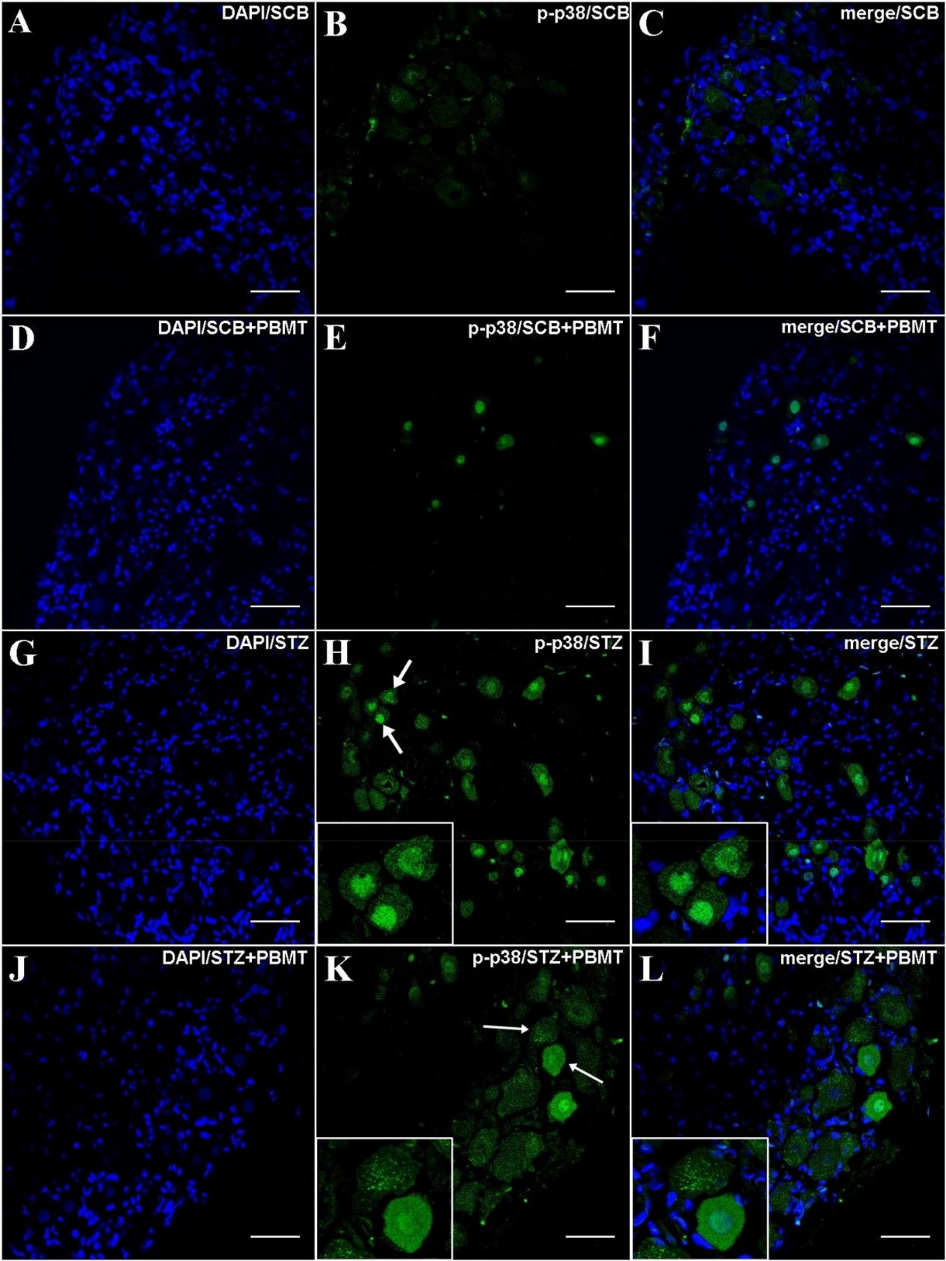
DRG histological micrographs of p-p38 MAPK by confocal microscopy. DRG transversal 14 µm-thick sections were submitted to IF protocol for staining endogenous p38 phosphorylation in Thr180 and Tyr182 (**B**,**E**,**H**,**K**). DAPI for nuclear staining is shown in (**A**,**D**,**G**,**J**). Merge (DAPI + p-p38) images are also shown in (**C**,**F**,**I**,**L**). In (**H**) and (**K**), white arrows point to the zoomed-in areas. Details are shown in the left corner of (**H**,**I**,**K**,**L**). Magnification: ×40; scale bars: 50 µm.

The immunofluorescence (IF) experiments also found a higher increase in the expression of activated MAPK, especially p-p38, in the STZ group (Fig. 5H,I). The fluorescence regarding p-38 phosphorylation in hyperalgesic rats was reduced by the application of PBMT in the DRG region, as shown in the STZ + PBMT group, in which there was a portion of p-38 activation but in a lower number of neurons in comparison to the STZ group (Fig. 5K,L). For both STZ and STZ + PBMT groups, the activation of p38 was highly concentrated in the nuclei of the DRG afferent neurons, as shown by DAPI co-staining (Fig. 5I,L in detail). Besides the lower number of p-p38 positive neurons in the STZ + PBMT group, it was also possible to observe a higher intensity of fluorescence scattered in neurons cytoplasm with some vesicle-like conformations (Fig. 5K,L in detail). However, the meaning of this difference compared with the hyperalgesic non-PBMT rats (STZ group) is unclear.

**Figure 6:**
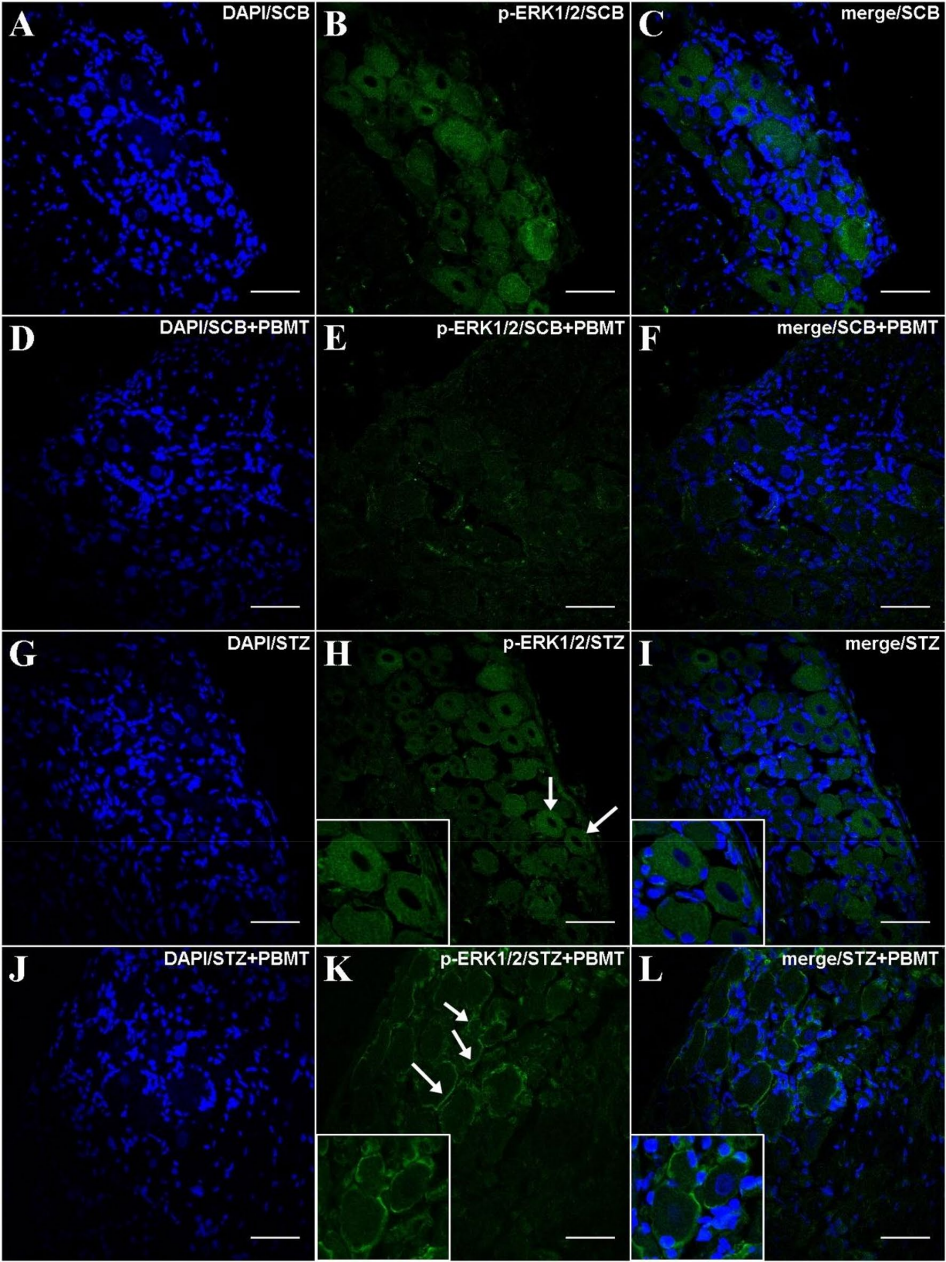
DRG histological micrographs of p-ERK1/2 MAPK by confocal microscopy. DRG transversal 14 µm-thick sections submitted to IF protocol for staining ERK1/2 phosphorylation in Thr202 and Tyr204 (p44/p42) (**B**,**E**,**H**,**K**). DAPI for nuclear staining is shown in (**A**,**D**,**G**,**J**). Images of merge (DAPI + p-ERK1/2) are also shown in (**C**,**F**,**I**,**L**). In (**H**) and (**K**), white arrows point to the zoomed-in areas. Details are shown in the left corner of (**H**,**I**,**K**,**L**). Magnification: ×40; scale bars: 50 µm.

Similarly to the gene expression results, low phosphorylation of ERK1/2 protein was detected. However, for both SCB + PBMT and STZ + PBMT groups (Fig. 6E,K, respectively), the levels of fluorescence-related p-ERK1/2 activation were even lower in comparison to SCB and STZ groups (Fig. 6B,H, respectively). An interesting observation regarding the STZ + PBMT group is the presence of p-ERK1/2 accrued in the vicinity of the plasma membrane, as shown in Fig. 6K,L (in detail).

**Figure 7:**
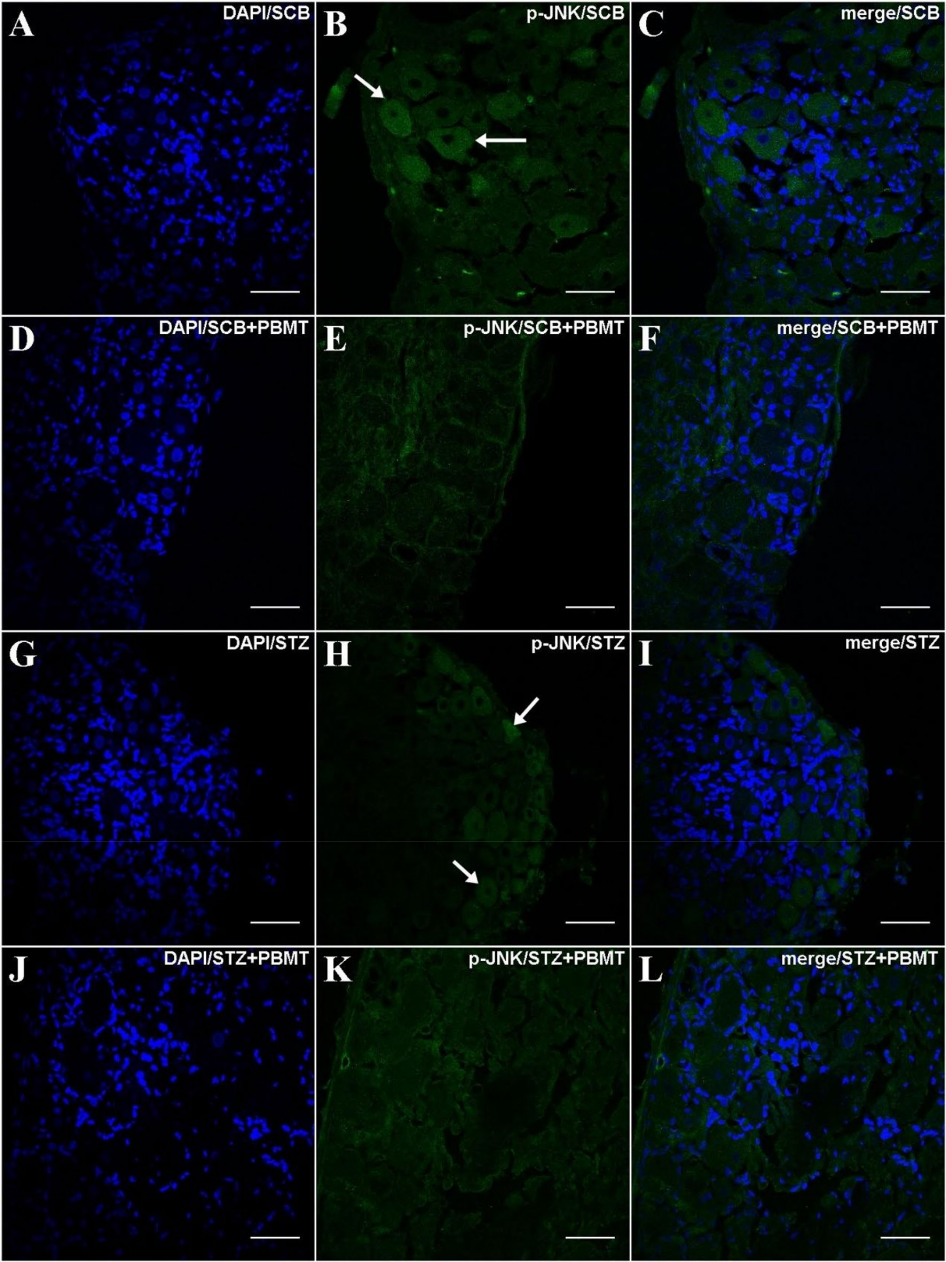
DRG histological micrographs of p-JNK MAPK by confocal microscopy. DRG transversal sections were 14 µm in thickness and submitted to IF protocol for staining of endogenous p-JNK phosphorylation in Thr183 and Tyr185 (p46/p54) (**B**,**E**,**H**,**K**). DAPI for nuclear staining is shown in (**A**,**D**,**G**,**J**). Merge (DAPI + p-JNK) images were also shown in (**C**,**F**,**I**,**L**). In (**B**) and (**H**), arrows point to diffused staining in the cytoplasm. Magnification: ×40; scale bars: 50 µm.

In line with ERK1/2 phosphorylation, the application of PBMT in the DRG region decreases the p-JNK expression independently of hyperalgesia (SCB + PBMT and STZ + PBMT groups; Fig. 7E,K, respectively). Rats that did not submit to PBMT presented a basal expression of p-JNK, observed in a diffused way in the cytoplasm (SCB and STZ groups; Fig. 7B,H, respectively).

It has been demonstrated that MAPK activation is associated with allodynia and hyperalgesia in different disease conditions^93–95^. The most studied member of the MAPK family, p38-MAPK, is typically activated by extracellular stress and proinflammatory cytokines, with a prominent role in the inflammatory process once there is a significant reduction in inflammation after the systemic administration of p38 pharmacological inhibitor^96^. Rats, by 8–12-weeks after STZ treatment, showed activation of p38, JNK, and ERK1/2 in L4-L5 DRG, although with delayed activation of JNK, relative to p38 and ERK1/2^41^. It has been suggested that TNF-α and IL-1β play a key role in developing and maintaining pain statuses after peripheral nerve injury^97^. Once p38 regulates TNF-α and IL-1β biosynthesis in DRG, both reductions can culminate in anti-inflammatory effects with a positive reflex in analgesia^97^. Therefore, our data showed that PBMT, a photophysical and photochemical effector of cell events, promotes a reduction in TNF-α and IL-1β associated with less activation (phosphorylation) of p38, which can help explain the analgesic effect of the therapy.

Although our data demonstrated a decrease, even slight, of MAPK activation by PBMT, the laser therapy (He–Ne; 632.8 nm; 4.5 mW; 3 s each 1.8 mm × 1.8 mm square) studied in tissue culture of skeletal muscle cells demonstrated an increase of ERK1/2, and no effect over p38 and JNK expression^98^. Also, laser irradiation (Er: YAG; 2.94 µm; fluence between 0.7 and 17.2 J/cm^2^) in osteoblast cell cultures demonstrated activation of ERK1/2 and no effect on p38 and JNK expression^99^. So, the effect of PBMT on the expression of MAPKs may depend on the tissue involved in this therapy.

It has been suggested that p38 activation in the DRG is initiated by retrograde transport of nerve growth factor (NGF) release from peripheral tissue, when inflamed, for example. It increases the translation and the transport of TRPV1 (a polymodal receptor channel) to the peripheral nociceptor terminal, contributing to the maintenance of heat pain sensitivity. In addition, p-p38 was found mainly in small neurons of the DRG, suggesting a higher activation in type-C sensory fibers^100^.

### The p-p38 staining is co-localized to TRPV1 in DRG

**Figure 8:**
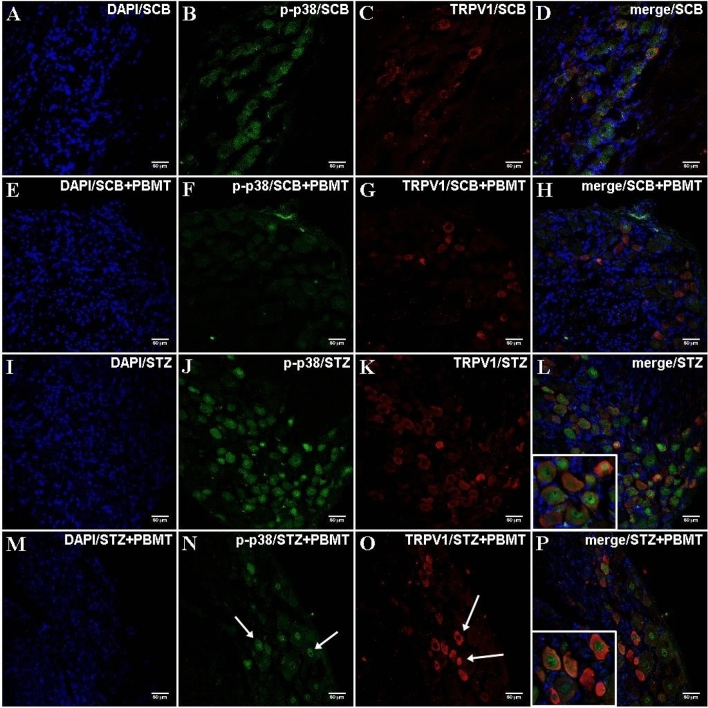
DRG histological micrographs of p-p38 MAPK and the co-staining of TRPV1 by confocal microscopy. DRG transversal sections were made in 14 µm thickness and submitted to IF protocol for staining endogenous p38 phosphorylation in Thr180 and Tyr182 (**B**,**F**,**J**,**N**) and TRPV1 (**C**,**G**,**K**,**O**). DAPI for nuclear staining is shown in (**A**,**E**,**I**,**M**). Merge (DAPI + p-p38 + TRPV1) images are also shown in (**D**,**H**,**L**,**P**). STZ + PBMT group showed moderate staining for p-p38 MAPK (**N**; white arrows) and increased staining for TRPV1 (**O**; white arrows). Details are shown in the left corner of (**L**) and (**P**). Magnification: ×40; scale bars: 50 µm.

Complementarily to the IF staining of p-p38 in DRG, it was further analyzed whether its expression was involved in a particular type of nerve fibers, i.e., type-C fibers (small DRG neurons; unmyelinated; TRPV1^+^). TRPV1^+^ fibers were detected in DRG sections of all experimental groups. They were classified as type-C, with higher intensity of fluorescence and occurrence in the STZ and STZ + PBMT groups (Fig. 8K,O, respectively). The co-staining of p-p38 and TRPV1^+^ fibers was widely distributed (Fig. 8L,P, respectively), with a prevalence of TRPV1^+^ signal (red) over p-p38 (green) in the STZ + PBMT group, as shown in Fig. 8P (in detail).

There is a correlation between TRPV1 and MAPK because the Ca^2+^ entry through TRPV1 leads to ATP release, P2Y2 purinergic receptor activation, and the transactivation of the epidermal growth factor receptor (EGFR). It has been described that the increase in [Ca^2+^]_i_ and the binding of ATP to P2Y2 upregulate the intracellular IP3 via phospholipase C (PLC). The upregulation of IP3 leads to the opening of store-operated channels (SOC), which causes Ca^2+^ release from the endoplasmic reticulum (ER). The previous TRPV1-mediated EGFR transactivation prompts Ras/Raf/MAPK signaling^101^.

The cutaneous tissue is innervated by TRPV1^+^ nociceptive nerve endings^102^, easily exposed to sunlight, and activated by ultraviolet (UV) light. UV light activates Ca^2+^ influx and non-selective cationic current in immortalized human keratinocytes (HaCaT cells)^103^. This activation was suppressed by capsazepine, a TRPV1 antagonist, thus showing an interaction between the light and TRPV1 channels^103^. In this sense, Wang et al.^104^ showed that PBMT through a 980 nm laser device (3 J/cm^2^; continuous wave) induced a thermal effect and, consequently, TRPV1 activation in adipose-derived stem cells. Our data from DRG suggested that PBMT increases the expression of TRPV1 in diabetic hyperalgesic rats, although, as described below, the PBMT decreased the Ca^2+^ influx in the DRG neurons culture. However, further studies should be done to investigate better the involvement of TRPV1 in the anti-hyperalgesic effect of PBMT applied in the DRG region of hyperglycemic rats.

### PBMT influenced Ca^2+^ dynamics in DRG-cultured neurons

**Figure 9:**
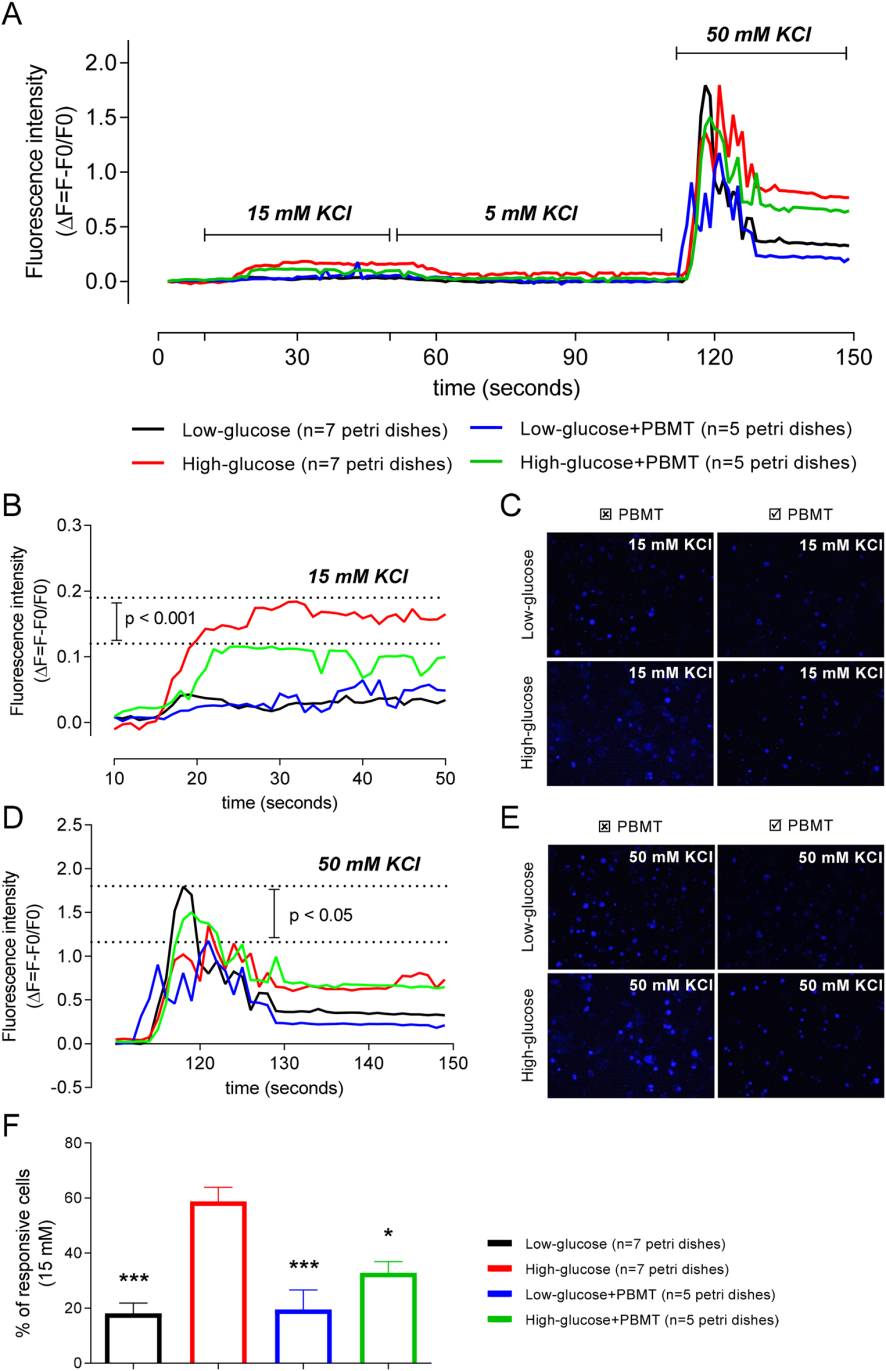
PBMT decreases the calcium dynamics of DRG neurons increased by hyperglycemia. Fluorescence intensity (ΔF = F − F0/F0) was analyzed in DRG neurons cultivated in low- (black line) or high-glucose (red line) media for 24 h and exposed to PBMT (green and blue lines) right before the test. (**A**) General representation of fluorescence intensities (ΔF = F − F0/F0) after extracellular stimuli with 5 mM (basal), 15 mM (intermediary), and 50 mM (high) of KCl; ↑[K^+^]_e_ was used to generate Ca^2+^ influx in DRG neurons incubated with FLUO-4 AM. (**B**) During the intermediary stimulus (15 mM KCl), there was a significant difference in the ΔF when comparing the High-glucose and High-glucose + PBMT groups (comparison was done considering the ΔF delimited by the horizontal dotted lines); Two-way ANOVA followed by Bonferroni posthoc test. (**C**) Snap-acquired images of the DRG cell culture during the time-lapse, right after the stimulus with 15 mM KCl. (**D**) At the high stimulus (50 mM KCl), there was a significant difference between the Low-glucose and Low-glucose + PBMT groups. (**E**) Snap-acquired images of the DRG cell culture during the time-lapse, right after the stimulus with 50 mM KCl. (**F**) Percentage (%) of responsive cells during the 15 mM stimulus; in the High-glucose group, about 60% of all responsive cells, i.e., cells with increased fluorescence during the 50 mM KCl stimulus (positive control), also responded to the intermediary stimulus (15 mM KCl), being statistically different from the other groups. Data are expressed as mean ± S.E.M.; symbols (*) and (***) mean p < 0.05 and p < 0.001, respectively, in comparison to the High-glucose group; One-way ANOVA followed by Bonferroni posthoc test.

The activation of the MAPK pathway and its modulation by PBMT seems to be related to the Ca^2+^ dynamics in the DRG neurons. The increase in the fluorescence intensity of FLUO-4 AM-stained DRG neurons was observed after the stimuli with 15 mM KCl and, especially, 50 mM KCl for all the groups (Fig. 9A). Remarkably, the increased fluorescence regarding the Δ[Ca^2+^]_i_ after a 15 mM KCl stimulus was mainly observed in neurons previously maintained in the hyperglycemic medium (High-glucose group) (Fig. 9B,C). In contrast, DRG neurons kept in a hyperglycemic medium (55 mM glucose) and exposed to PBMT (High-glucose + PBMT group) showed a significant (p < 0.001) decrease in fluorescence intensity during the 15 mM KCl stimulus compared to the High-glucose group (Fig. 9B,C). During the 50 mM KCl stimulus, which was used as a control for the stimulus of Ca^2+^ influx in responsive neurons, the main difference (p < 0.05) was observed between the fluorescence intensity of the Low-glucose group compared to the Low-glucose + PBMT group (Fig. 9D,E). When the 50 mM KCl stimulus ceased, the fluorescence intensity decreased in both hyperglycemic groups, although it did not return to basal levels (Fig. 9D,E).

Beyond the fluorescence intensity (Δ[Ca^2+^]_i_) we also quantified the percentage (%) of 15 mM KCl-responsive cells about the total number of neurons responsive to 50 mM KCl (Fig. 9F). The % of neurons responsive to the 15 mM KCl stimulus was higher in the High-glucose group compared to the Low-glucose (p < 0.001) and the High-glucose + PBMT groups (p < 0.05). This data suggests that PBMT reduces the responsiveness of the DRG neurons increased by hyperglycemia. Thus, it is plausible to hypothesize that one of the possible analgesic mechanisms of PBMT in the DRG region is, at the same time, increasing the TRPV1 expression in DRG neurons via IL-1β-induced p38 MAPK and desensitizing the TRPV1^+^ neurons by decreasing Ca^2+^ influx.

## Conclusion

PBMT might represent a novel therapeutic approach for treating diabetic neuropathic hyperalgesia, based on its beneficial photophysical and photochemical effects against the functional, molecular and cellular alterations observed in such disease, ruled by the MAPK pathway and calcium dynamics.

## Materials and methods

### Ethics statement and animals

All experiments were approved by the institutional Committee for Ethics in Animal Use (CEUA/UNICAMP, permit number 5337-1/2019) and followed the ARRIVE guidelines. The experiments were also performed according to the Brazilian National Council for Animal Experimentation Control (CONCEA) and the Brazilian College of Animal Experimentation (COBEA) guidelines. Male Lewis rats (LEW/HsdUnib, Harlan, USA, 1996), 4–8-week-old, weighing 200–250 g, provided by the University's Multidisciplinary Center for Biological Research (CEMIB) were used. Rats were maintained in plastic cages with sawdust bedding (changed three times a week), in some four per cage, and received food (commercial chow for rodents) and filtered water ad libitum, in a temperature- and humidity-controlled room under 12/12 h dark/light cycle.

Rats were randomly divided into five experimental groups, consisting of eight rats (n = 8) per group: Naïve (intact rats; received no injection and no PBMT); SCB (received five doses of the vehicle, 0.1 M sodium citrate buffer, and no PBMT); STZ (received five doses of STZ, 25 mg/kg per dose, and no PBMT); SCB + PBMT (received five doses of the vehicle and were submitted to PBMT); STZ + PBMT (received five doses of STZ and were submitted to PBMT). All efforts were made to minimize the number of rats and their suffering.

### T1D was induced through multiple low doses of streptozotocin (STZ)

The T1D induction was performed according to previous studies performed by our group^66,71–74^, consisting of a low dose of STZ (25 mg/kg) (N-[Methylnitrosocarbamoyl]-α-d-glucosamine; Sigma-Aldrich^®^, St. Louis, MO, USA]), diluted in the vehicle (0.1 M sodium citrate buffer, SCB [pH 4.5]) and injected intraperitoneally (i. p.) once a day, during five consecutive days (STZ and STZ + PBMT groups). The same volume of vehicle was injected daily in control animals (SCB and SCB + PBMT groups), ranging from 50 to 62.5 µL, according to rats' weight. Blood glucose measurements from the tail vein were performed with an Accu-Chek^®^ Sensor Comfort (Roche Diagnostics^®^, Germany). The development of hyperglycemia and the rats' weight were monitored on days 0, 7, 14, 21, 24, and 28 after starting the STZ or vehicle injections.

### The electronic von Frey test

The hyperalgesia was measured through the mechanical withdrawal thresholds, determined by applying the electronic von Frey test (Insight^®^, Ribeirão Preto, SP, Brazil). We used a polypropylene pipette tip adapted to a hand-held force transducer with crescent pressure in the plantar surface of the right and left rats' hind paws. The equipment automatically transduces the pressure applied to the paw mid-plantar surface into gram-force (g) once the paw is withdrawn. Rats were randomly placed into individual plastic cages with a metal mesh floor, followed by 30 min of acclimatization before the test. Electronic von Frey tests were applied to all experimental groups at 0, 7, 14, 21, 24, and 28 days after starting the STZ or vehicle injections, always in the mid-morning period, by a blind examiner to the groups and treatment. For the STZ + PBMT group, an additional analysis was done 19 days after starting the STZ injections to select only hyperalgesic rats for the PBMT exposure.

### CatWalk dynamic motor function analysis

CatWalk walking track test (Noldus Inc., Wageningen, Netherlands) consists of an illuminated walkway glass floor with a high-speed video camera (Gevicam GP-3360; GEViCAM Inc., Milpitas, CA, USA) equipped with a wide-angle lens (6.0 mm; DF6HA-1B, Fujinon Corp., China). The camera was positioned underneath the walkway at 56 cm, and the CatWalk™ XT 10.6 software automatically recorded the paw prints as the animal crossed the pathway in a calibrated 20 × 10 cm length lane. The green LED plus a red-illuminated background creates a contrast in the glass floor according to animal steps. CatWalk XT configurations were set up according to the parameters used by Vieira et al.^74^. For the current study we considered the average between the hind paws (right hind paw [RH] and left hind paw [LH]) regarding the Maximum Contact Area (cm^2^), Print Area (cm^2^), and Stride Length (cm), which were analyzed at 0, 7, 14, 21, 24, and 28 days after starting STZ or vehicle injections, always in the afternoon with the room lights switched off. Each rat performed 3 runs every analysis period, thus completing 24 runs per group per period.

### Photobiomodulation therapy (PBMT) using 904 nm wavelength irradiation

Rats from SCB + PBMT and STZ + PBMT groups were submitted to PBMT for eight days (from the 21st to the 28th day after STZ or vehicle injections), once a day, always in the morning. For the STZ + PBMT group, only hyperalgesic rats were considered to receive the laser treatment since, in our DN model, about 20% of the rats receiving STZ injections did not become hyperalgesic (data not shown). PBMT was performed with an Endophoton LLT1307 (KLD Biosistemas Equip. Elet. Ltda^®^, Amparo, SP, Brazil) class IIIB laser device. Rats were anesthetized with Isoflurane (3%) (Cristália^®^, Itapira, SP, Brazil), and the laser irradiation was applied directly on the rat dorsal shaved skin at a single point between L4-L5 spine levels, bilaterally. PBMT parameters are described in Table 1.

**Table 1:** PBMT parameters.

Laser	Wavelength	Power	Output	Irradiance
GaAs	904 nm	70 mW	0.001 cm^2^	7000 mW/cm^2^

Emission	Total energy	Time	Contact	Treated area
Continuous	2.03 J	29″ per point	Direct	~ 1.0 cm^2^

GaAs = Gallium-Arsenide; nm = nanometers; mW = milliWatts; J = Joule; (″) = seconds; cm^2^ = square centimeters; mW/cm^2^ = milliWatts per square centimeter.

### DRG collection, tissue preparation, and homogenization

For the analyses of inflammatory cytokines levels and MAPK gene expression, rats were anesthetized under Isoflurane (3%) (Cristália^®^) and humanely euthanized. After dissection, L4 and L5 DRG were collected, snap-frozen in liquid nitrogen (− 196.15 °C), and stored at − 80 °C for posterior homogenization. To the DRG samples for ELISA immunoassays, were added RIPA Lysis and Extraction Buffer (Thermo Scientific™, Waltham, MA, USA), containing sodium orthovanadate, protease inhibitor, and PMSF (phenylmethanesulfonyl fluoride) (Thermo Scientific™). Samples were placed in a FastPrep^®^ homogenizer (MP Biomedicals™, Santa Ana, CA, USA) and then were shaken at 4 °C (5 × 20 s) between 5 min intervals. After that, homogenized DRG were kept under continuous agitation for 3 h at 4 °C and then centrifuged at 12,000 rpm for 15 min at 4 °C. The resulting supernatant was transferred to a new tube. Protein concentrations were measured through the Bradford assay^105^.

For MAPK gene expression through real-time RT-qPCR, DRG were fragmented and homogenized in TRIzol^®^ (Invitrogen Life Technologies™, Carlsbad, CA, USA) (1 mL/mg) for isolating the total RNA, according to the manufacturer's instructions. To the homogenate were added 0.2 mL of chloroform (Sigma-Aldrich^®^), and after 3 min of resting at room temperature, it was centrifuged at 12,000 rpm for 15 min at 4 °C. The aqueous phase was transferred to a new tube, in which 0.5 mL of isopropanol was added. After a new centrifugation, the pellet was washed with 75% ethanol, and the total RNA was resuspended in UltraPure™ DEPC-treated water (Thermo Scientific™). Total DRG RNA was quantified using an ultra-low-volume spectrophotometer (Epoch Microplate Spectrometer, BioTek Instruments Inc., Winooski, VT, USA).

For DRG immunofluorescence (IF), rats were anesthetized with ketamine (85 mg/kg, i. p.) and xylazine (10 mg/kg, i. p.) and then exsanguinated via cardiac perfusion (through the ascending aorta) with saline solution (0.9% NaCl, 200 mL). After exsanguination, rats were perfused with 4% paraformaldehyde (PFA, pH 7.4, 4 °C, 300 mL). Then, L4 and L5 DRG were collected and post-fixed in 4% PFA overnight at 4 °C, followed by 48 h in 30% sucrose at 4 °C. Individual DRG were embedded in Tissue-Tek^®^ O.C.T. compound (Sakura^®^ Finetek, CA, USA), and 14 µm non-serial sections were made on a cryostat (Leica Biosystems, Wetzlar, Germany) using gelatinized slides.

### ELISA immunoassay for measuring the cytokines concentrations (IL-1β, TNF-α, IL-6, CINC-1 and IL-10)

For IL-1β, TNF-α, IL-6, and CINC-1 were used 96-well-plates through the DuoSet^®^ ELISA kit (R&D Systems, Minneapolis, MN, USA), and the results were expressed in picograms per milliliter (pg/mL). For the IL-10, concentrations were measured through the RayBio^®^ Rat IL-10 ELISA kit (#ELR-IL10) (RayBiotech, Peachtree Corners, GA, USA), and the results were expressed in picograms per milliliter per milligram (pg/mL/mg) of tissue. The manufacturer's instructions were followed for both ELISA kits. The absorbance was determined at 450 nm using an Asys UVM 340 microplate reader (Biochrom Ltd., Cambridge, UK), and the results were obtained by comparing the optical density to the standard-curve densities.

### Real-time quantitative RT-PCR for MAPK gene expression

500 ng of total RNA extracted from L4 and L5 DRG were subjected to cDNA synthesis (SuperScript™ VILO™ cDNA Synthesis Kit, Invitrogen Life Technologies™) according to the manufacturer's protocol. Specific primers for p38, ERK1/2, JNK, Arfgef1, and Serpinb6 genes were designed with the "*Pick Primers*" tool, available on NCBI/Primer-blast (http://www.ncbi.nlm.nih.gov/tools/primer-blast) and synthesized commercially. The amplicons were set to less than 200 base pairs, and dimers, cross-dimers, and hairpins were eliminated during primers design (or were kept to a minimum). Primer sequences are described in Table 2. The annealing temperature was set at 60 °C and the GC (guanine-cytosine) content was 50–55%.

**Table 2:** 5'-3'gene sequences.

Gene	Sequence	GenBank
p38^a^	FW: GGCTGACATAATCCACAGGG	NM_031020.2
	RV: CCGGTCATTTCGTCATCAGT	
ERK1/2^a^	FW: TGTGTTCAGCTCAGACTTCC	NM_133283.1
	RV: CGTTTGATGAAGGCATGGTT	
JNK^a^	FW: TGCTACTTGCCAATCCCATC	NM_053829.2
	RV: AGATAACAGGGTGTCCGCTA	
Arfgef1^b^	FW: CAACAGGTTTAAAGCTCACGCA	NM_001277056.1
	RV: TCCTGTTCAGGTGGTTGTGA	
Serpinb6^b^	FW: GAGTCTAGGGTACGTTCTGCTG	NM_199085.2
	RV: TCCATGATGGTGAACCTGCCC	

*FW*  forward;* RV* reverse.

^a^Target genes; ^b^housekeeping.

The 2^−ΔΔCt^ method^106^ performed relative gene expression analysis to an internal control index. The reactions were carried out at the StepOne Plus Real-Time PCR (Applied Biosystems, Foster City, CA, USA) using SYBR Green as a fluorescent signal (Power SYBR™ Green PCR Master Mix, Applied Biosystems, USA) and 1:10 of the obtained cDNA from each sample.

### DRG immunofluorescence (IF) for detection of phosphorylated MAPKs

DRG sections were incubated in 0.1 M glycine for 30 min, followed by a blockage with 2% bovine serum albumin (BSA) and permeabilization in 0.2% Triton X-100 for 1 h at room temperature. For anti-phospho-p44/42 MAPK (ERK 1/2) and anti-phospho-SAPK/JNK, an additional step was done before the 2% BSA blocking, consisting of methanol 100% permeabilization at − 20 °C. Then, sections were incubated in 0.1 M PBS plus 0.1% Triton-100 and 1% BSA overnight in a humid atmosphere at 4 °C with the specific primary antibodies. The following antibodies were used: anti-phospho-p38 MAPK (Thr180/Tyr182) (D3F9) XP^®^ Rabbit mAb (1:500); anti-phospho-p44/42 MAPK (ERK 1/2) (Thr202/Tyr204) (D13.14.4E) XP^®^ Rabbit mAb (1:200); and anti-phospho-SAPK/JNK (Th183/Tyr185) (G9) Mouse mAb (1:400), all from Cell Signaling Technology^®^ (Danvers, MA, USA). After the incubation period, the sections were washed twice in the same incubation solution (without the antibodies) and then washed 5 times in 0.1 M PBS, 5 min each time. Sections were then incubated with the secondary antibodies (donkey anti-rabbit or anti-mouse Alexa 488, 1:1000, #A21206, Thermo Scientific™) diluted in the same primary antibody solution for 1 h at room temperature. After incubation, DRG sections were washed with 0.1 M PBS five times for 5 min.

Nuclei were stained with 4′,6-Diamidino-2-phenylindole dihydrochloride (DAPI, 0.25 µg/mL, D9542, Sigma-Aldrich^®^) diluted in 0.1 M PBS, for 10 min at room temperature and sections were coverslipped using Vectashield^®^ (Vector Laboratories, Burlingame, CA, USA). Negative controls were prepared without incubation in primary antibodies to confirm non-specific binding. The slides were first examined in an epifluorescence inverted Leica DMi6000 B microscope coupled with a DFC360 FX camera and a Leica fluorescent light source CTR7000HS (Leica Microsystems). After the confirmation of positive fluorescence, the final images were obtained in a Zeiss Axio Observer Z1 LSM780-NLO (Carl Zeiss AG, Oberkochen, Germany) confocal laser-scanning microscope, with the aid of the EC Plan-Neofluar 20x/0.50 Dry and EC Plan-Neofluar 40×/1.30 Oil DIC objective lens.

### DRG primary cell culture

Primary cell culture of DRG neurons was performed according to the protocol described by Linhart et al.^107^ and modified by our research group as published by Manzo et al.^108^ and do Prado et al.^73^. Healthy male Lewis rats (LEW/HsdUnib, Harlan, USA, 1996) weighing about 200 g, 4 weeks old, were used. Rats were humanely euthanized under deep anesthesia (3% Isoflurane; Cristália^®^) followed by decapitation. Sixteen to twenty thoracic and lumbar DRGs were collected and placed in Hank's Balanced Salt Solution (HBSS, containing 10 mM of HEPES). Then, cells were dissociated by incubation with HBSS containing 0.28 U/mL of collagenase type II for 60 min at 37 °C followed by 6 min in 0.25 mg/mL trypsin. For inhibition of the trypsin action, cells were washed twice in DMEM supplemented with fetal bovine serum (FBS; 10%), 50 U/mL of penicillin, and 50 mg/mL of streptomycin. Mechanically-dissociated cells were plated on laminin and poly-d-lysine-coated coverslips and maintained at 37 °C and 5% CO_2_. All cell culture supplies were purchased from Sigma-Aldrich^®^ except FBS, purchased from Vitrocell Embriolife (Campinas, SP, Brazil).

### Calcium imaging

After completion of 24 h incubation under normoglycemic (5.5 mM of glucose) or hyperglycemic (55 mM of glucose) cell medium, sensory DRG neurons were exposed to PBMT with the same parameters described in Table 1 but with indirect contact through the "*swiping motion*", where the laser probe output was kept in a distance of 1 cm far from the cell medium. Immediately after the irradiation, DRG-cultured neurons were incubated in HBSS containing 10 µM of FLUO-4, AM, and 1% PowerLoad (Thermo Scientific™) for 40 min, protected from the light at 37 °C and 5% CO_2_. Coverslips were inserted into a perfusion chamber (Warner Instruments, Holliston, MA, USA) and placed on an inverted Leica DMi6000 B microscope coupled to a DFC360 FX camera and a Leica fluorescent light source CTR 7000 HS (480 nm excitation, 527/30 nm suppression filters) (Leica Microsystems). A computer-controlled valve system (Warner Instruments) was used for cell perfusion with different KCl molarities [5 mM (basal), 15 mM (partial), and 50 mM (maximum)], and the flow rate was set at 5 mL/min (complete change of chamber volume every 2 s)^73,108^. Images were taken at the rate of one image/s, and the values of fluorescence intensity (Δ[Ca^2+^]_i_) were normalized by ΔF/F0, where ΔF equals the final fluorescence (F) minus the basal fluorescence (F0). Data were also presented as the % of 15 mM KCl-responsive cells about the total number of cells responsive to 50 mM KCl, in four distinct situations: (i) when cells were incubated in low-glucose; (ii) low-glucose plus PBMT; (iii) high-glucose or (iv) high-glucose plus PBMT.

### Statistical analysis

For comparisons between groups and treatment time, we used Two-way ANOVA followed by Bonferroni posthoc test. One-way ANOVA made other comparisons between three or more groups, followed by Bonferroni posthoc test. For comparisons between only two groups, we used an unpaired Student *t-test*. p < 0.05 (*), p < 0.01 (**), and p < 0.001 (***) were considered statistically significant. All statistical tests were performed on the GraphPad Prism^®^ software, versions 5 and 7. Numerical values were expressed as mean ± standard error of the mean (SEM).

## Data Availability

The data that support the findings of this study are available upon request from the corresponding author [CAP].
